# Molecular detection of *Anaplasma bovis*, *Ehrlichia canis* and *Hepatozoon felis* in cats from Luanda, Angola

**DOI:** 10.1186/s13071-018-2767-y

**Published:** 2018-03-20

**Authors:** Ana Cristina Oliveira, Maria Francisca Luz, Sara Granada, Hugo Vilhena, Yaarit Nachum-Biala, Ana Patrícia Lopes, Luís Cardoso, Gad Baneth

**Affiliations:** 1 Clínica Casa dos Animais, Luanda, Angola; 2 Center for Investigation Vasco da Gama (CIVG), Department of Veterinary Medicine, Vasco da Gama University School, Coimbra, Portugal; 3 Baixo Vouga Veterinary Hospital, Águeda, Portugal; 4 Animal and Veterinary Research Centre (CECAV), University of Trás-os-Montes e Alto Douro (UTAD), Vila Real, Portugal; 5 Koret School of Veterinary Medicine, The Hebrew University of Jerusalem, Rehovot, Israel; 6 Department of Veterinary Sciences, School of Agrarian and Veterinary Sciences (ECAV), UTAD, Vila Real, Portugal

**Keywords:** DNA sequencing, Feline vector-borne diseases, Polymerase chain reaction, Tick-borne pathogens

## Abstract

**Background:**

Molecular identification of tick-borne pathogen infection in cats from Africa is scarce. The presence of bacterial (*Anaplasma* and *Ehrlichia*) and protozoal (*Babesia* and *Hepatozoon*) agents was investigated in blood samples from 102 domestic cats from Luanda, Angola, by polymerase chain reaction and DNA sequencing.

**Results:**

Three cats (2.9%) were found infected with *Ehrlichia canis*, three (2.9%) with *Hepatozoon felis* and one (1.0%) with *Anaplasma bovis*. The prevalence of infections with one single agent was 4.9%, and that of infection with two agents (i.e. *E. canis* and *H. felis*) was 1.0%. In total, six cats (5.9%) were found infected with at least one of the detected tick-borne agents.

**Conclusions:**

This is the first report of *A. bovis*, *E. canis* and *H. felis* in cats from Angola. To the best of our knowledge, *A. bovis* is also being reported for the first time in domestic cats outside of Japan. Cats are at a low to moderate risk of being infected with tick-borne agents in Luanda.

**Electronic supplementary material:**

The online version of this article (10.1186/s13071-018-2767-y) contains supplementary material, which is available to authorized users.

## Background

Bacteria and protozoa transmitted by various arthropods including ixodid ticks cause several diseases in cats [1, 2]. Some of those vector-borne agents have a zoonotic character, i.e. they may be transmitted from animals and infect humans [3]. From a clinical point of view, vector-borne infections pose a diagnostic challenge because of their non-specific manifestations and often subclinical nature [4]. Molecular methods, namely the polymerase chain reaction (PCR) and DNA sequence analysis, are useful for the diagnosis of individual clinical cases as well as for epidemiological studies on tick- and other vector-borne agents [5, 6].

Infection with several species of tick-borne bacteria and protozoa has been described in felids from Africa [7–9], but no molecular data are available on tick-borne pathogens in cats from Luanda, Angola. In this study, agents of the genera *Anaplasma*, *Babesia*, *Ehrlichia* and *Hepatozoon* were surveyed in blood samples from 102 domestic cats from Luanda, Angola, by PCR and DNA sequencing.

## Methods

### Cats and samples

Domestic cats (*n* = 102) were sampled at “Casa dos Animais” veterinary clinic in Luanda, Angola, from May 2014 to February 2016. Available data are displayed by independent variables and their categories in Table 1. The age of cats ranged from 2.5 to 143 months (median: 12 months; interquartile range: 7.5–24).

**Table 1:** Prevalence of tick-borne pathogens in 102 cats from Luanda, Angola, as determined by PCR and DNA sequencing

Variable/Category	No. of cats tested (%)	Percentage (*n*) of infected cats	95% CI (%)
Gender	101		
Female	56 (55.4)	5.4 (3)	1.1–14.9
Male	45 (44.6)	6.7 (3)	1.4–18.3
Breed	96		
Mixed	92 (95.8)	6.5 (6)	2.4–13.7
Defined^a^	4 (4.2)	0.0 (0)	0.0–60.2
Age group	100		
Juvenile^b^	50 (50.0)	6.0 (3)	1.3–16.5
Adult^c^	50 (50.0)	6.0 (3)	1.3–16.5
Hair length	102		
Short	85 (83.3)	7.1 (6)	2.6–14.7
Medium or long	17 (16.7)	0.0 (0)	0.0–19.5
Housing	102		
Indoors	37 (36.3)	0.0 (0)	0.0–9.5
Outdoors or mixed	65 (63.7)	9.2 (6)	3.5–19.0
Contact with other animals	97		
No	20 (20.6)	0.0 (0)	0.0–16.8
Yes^d^	77 (79.4)	7.8 (6)	2.9–16.2
Travel^e^	102		
No	60 (58.8)	3.3 (2)	0.4–11.5
Yes	42 (41.2)	9.5 (4)	2.7–22.6
Ectoparasiticides	91		
No	84 (92.3)	6.0 (5)	2.0–13.3
Yes^f^	7 (7.7)	14.3 (1)	0.4–57.9
Fleas	102		
No	89 (87.3)	5.6 (5)	1.9–12.6
Yes^g^	13 (12.7)	7.7 (1)	0.2–36.0
Clinical status	102		
Apparently healthy	89 (87.3)	6.7 (6)	2.5–14.1
Sick^h^	13 (12.7)	0.0 (0)	0.0–24.7
Total	102 (100)	5.9 (6)^i^	2.2–12.4

*Abbreviations*: *95% CI* 95% confidence interval

^a^Comprising 2 Persian and 2 Siamese cats

^b^2.5–11.5 months

^c^12–143 months

^d^Including cats, dogs, rodents and/or birds

^e^Outside the province of Luanda

^f^Fipronil

^g^Not identified

^h^Clinical manifestations and laboratory abnormalities: anemia, anorexia/hyporexia, cough, cutaneous lesions, diarrhea, fever, leukocytosis, leukopenia, neurological disorders, ocular signs, thrombocytopenia, weight loss, vomiting

^i^*Anaplasma bovis* (*n* = 1), *Ehrlichia canis* (*n* = 3) and *Hepatozoon canis* (*n* = 3), including 1 cat co-infected with both *E. canis* and *H. felis*

Blood was collected in EDTA tubes and centrifuged, with two thirds of the plasma volume separated from cells and the remaining plasma frozen together with cells at -20 °C. DNA was extracted from the concentrated blood samples using a commercial kit (E.Z.N.A.® Blood DNA Mini Kit, Omega Bio-Tek, Norcross, GA, USA), according to the manufacturer’s instructions.

### DNA amplification and sequencing

All DNA samples were screened for the presence of *Anaplasma* and *Ehrlichia* spp. in duplicates by a real time PCR assay targeting a 123 bp fragment of the *16S* ribosomal RNA (rRNA) gene using the primers E.c 16S-fwd (5'-TCG CTA TTA GAT GAG CCT ACG T-3') and E.c 16S-rev (5'-GAG TCT GGA CCG TAT CTC AG-3'), as previously described [10]. PCR amplification was performed using the StepOnePlus real-time PCR thermal cycler (Applied Biosystems, Foster City, CA, USA) in a total volume of 20 μl containing 4 μl DNA, 400 nM of each primer, 10 μl Maxima Hot Start PCR Master Mix (2×) (Thermo Scientific, Epsom, Surrey, UK), 50 μM of SYTO9 solution (Invitrogen, Carlsbad, CA, USA) and sterile DNase/RNase-free water (Sigma, St. Louis, MO, USA). Initial denaturation for 5 min at 95 °C was followed by 40 cycles of denaturation at 95 °C for 5 s, annealing at 59 °C for 30 s, and a final extension at 72 °C for 20 s. Amplicons were subsequently subjected to a melt step with the temperature raised to 95 °C for 10 s and then lowered to 60 °C for 1 min. The temperature was then raised to 95 °C at a rate of 0.3 °C per second. Amplification and melt profiles were analyzed using the StepOnePlus software v.2.2.2 (Applied Biosystems). Positive samples were further analyzed by a conventional PCR using the primers EHR16SD (5'-GGT ACC YAC AGA AGA AGT CC-3') and EHR16SR (5'-TAG CAC TCA TCG TTT ACA GC-3') [11] targeting a 345 bp fragment of the *16S* rRNA gene. PCR was performed using a programmable conventional thermocycler (Biometra, Göttingen, Germany). The reaction was done using PCR-ready High Specificity mix (Syntezza Bioscience, Jerusalem, Israel) in a total volume of 25 μl including 500 nM of each primer and sterile DNase/RNase-free water (Sigma). Amplification was performed with an initial denaturation at 95 °C for 5 min, followed by 35 cycles of denaturation at 95 °C for 30 s, annealing at 57 °C for 30 s, and a final extension at 72 °C for 30 s. After the last cycle, the extension step was continued for a further 5 min. PCR products were electrophoresed on 1.5% agarose gels stained with ethidium bromide and evaluated under UV light for the size of amplified fragments by comparison to a 100 bp DNA molecular weight marker. DNA extracted from an *Ehrlichia canis* cell culture and from the blood of a dog infected with *Anaplasma platys* confirmed by PCR and sequencing were used as positive controls in all reactions.

Testing for the presence of *Babesia* and *Hepatozoon* spp. was performed by screening all DNA samples by a conventional PCR using the primers Piroplasmid-F (5'-ATA CAT GAG CAA AAT CTC AAC-3') and Piroplasmid-R (5'-CTT TCG CAG TAG TTY GTC TTT AAC AAA TCT-3'), which amplify a 350–400 bp fragment of the *18S* rRNA gene of *Hepatozoon* spp. and *Babesia* spp. [12]. The reaction was done as above except for the annealing temperature, which was 64 °C. DNA samples extracted from a dog infected with *Hepatozoon canis* and from another dog infected with *Babesia vogeli* confirmed by PCR and sequencing were used as positive controls.

DNA from the blood of a laboratory-bred pathogen free dog was used as a negative control. Non-template control reactions were done using the same procedures and reagents described above, but without DNA added to the PCR to rule out contamination and non-specific reactions. Negative uninfected dog DNA, and non-template DNA controls were used in each run for all pathogens.

All positive PCR products were sequenced at Hy Laboratories Ltd. (Rehovot, Israel) using the BigDye terminator v.1.1 Cycle Sequencing Kit (Applied Biosystems) on the ABI PRISM 3730xl DNA Analyzer. Raw data was analyzed using DNA Sequencing Analysis Software v.5.4. DNA sequences were evaluated with the ChromasPro software version 2.1.1 (Technelysium Pty Ltd., South Brisbane, QLD, Australia) and compared for similarity with sequences available in GenBank, using the BLAST program (http://www.ncbi.nlm.nih.gov/BLAST/). Species identity was determined as the closest BLAST match of at least 97–100% identity to an existing GenBank accession [13–15].

### Data analysis

The Chi-square test (CST) and Fisher’s exact test (FET) was used to compare proportions of positivity, considering a probability (*P*) value < 0.05 as statistically significant. Exact binomial 95% confidence intervals (CI) were established for proportions. Analyses were done using the WinEpi, IBM SPSS Statistics 20 and StatLib softwares. Assuming a default expected prevalence of 50% and a confidence level of 95%, a sample size of 102 units involves an absolute error of 9.7% [16].

## Results

Three cats (2.9%; 95% CI: 0.6–8.3%) were found infected with *E. canis*, three (2.9%; 95% CI: 0.6–8.3%) with *Hepatozoon felis* and one (1.0%; 95% CI: 0.0–5.3%) with *Anaplasma bovis*. The prevalence of infection with one single agent was 4.9% (95% CI: 1.6–11.1%), and that of coinfection with two agents (i.e. *E. canis* and *H. felis*) was 1.0% (95% CI: 0.0–5.3%) (FET: *P* = 0.097). In total, six cats (5.9%; 95% CI: 2.2–12.4%) were found infected with at least one of the detected tick-borne agents (Table 1). No statistically significant differences were found between positivity to any one of the three detected agents, either in single or coinfection, between the categories of gender, breed, age group, hair length, housing, contact with other animals, travel, ectoparasiticides, fleas and clinical status (Table 1). The identification of feline tick-borne agents according to the similarity of their amplified sequences with those available in the GenBank database is displayed in Table 2 (see also Additional file 1: Table S1).

**Table 2:** Tick-borne pathogens from the six positive cats and their similarity with sequences deposited in the GenBank database

Primer	Closest GenBank accession	Percent identity (no. of cats)^a^	Agent	Sample ID	New GenBank accessions
E.c 16S-fwd/E.c 16S-rev	KX987326	100 (1)	*Ehrlichia canis*	017	na
	KX987326	100 (1)	*Ehrlichia canis*	053	na
	KX987326	99 (1^b^)	*Ehrlichia canis*	002	na
E.c 16S-fwd/E.c 16S-rev + EHR16SD/EHR16SR	KY425447	99 (1)	*Anaplasma bovis*	026	MG431981
Piroplasmid-F/Piroplasmid-R	KY649442	100 (1)	*Hepatozoon felis*	063	MG386484
	KY649443	100 (1)	*Hepatozoon felis*	056	MG386483
	KY649443	99 (1^b^)	*Hepatozoon felis*	002	MG386482

*Abbreviations*: *na* not available, as sequences < 200 bp cannot be deposited in GenBank (see also Additional file 1: Table S1)

^a^Only sequence identity ≥ 97% was considered as positive

^b^Same animal

All of the molecularly detected agents were found in apparently healthy cats. The cat found infected with *A. bovis* and one of the three other cats infected with *H. felis* had never travelled outside the province of Luanda. The three cats found infected with *E. canis*, including one cat coinfected with *H. felis*, had travelled out of Luanda.

## Discussion

This is the first description of *A. bovis*, *E. canis* and *H. felis* in cats from both Luanda and Angola. Furthermore, and to the best of our knowledge, *A. bovis* is also being reported for the first time in domestic cats outside of Japan [17]. Results of the present study suggest that domestic cats in Luanda are at a low to moderate risk of being infected with one or more of these three tick-borne agents.

Although several vector-borne agents cause morbidity and mortality in domestic feline populations, the importance of some of them as a cause of disease has not yet been clearly determined [18]. All of the molecularly detected agents were found in apparently healthy animals, a situation which is in agreement with the generally subclinical nature of these infections [2]. Nevertheless, further studies are necessary to determine the real impact of these agents in cats.

*Anaplasma bovis* infection has been reported from several countries and in a few vertebrate species [17], mainly affecting cattle, with fever, anemia, weight loss, lymphadenopathy, abortion and death. However, subclinical infections have also been documented [19]. The first detection of *A. bovis* in domestic felines was reported in two cats from the Ehime Prefecture in western Japan [17]. The two cats had stomatitis and coinfection with the feline immunodeficiency virus (FIV); and one of the cats also had anorexia, diarrhea and fever, and co-infection with the feline leukemia virus. Although both those cats had stomatitis, the association between *A. bovis* infection and clinical illness could not be established, because stomatitis is a common finding in cats with FIV infection [17]. *Anaplasma bovis* was additionally detected in blood from Tsushima leopard cats (*Prionailurus bengalensis euptilurus*) from Japan [20], in the same leopard cat subspecies from Korea [21] and in one *Haemaphysalis longicornis* nymph obtained from an Iriomote (leopard) cat (*P. bengalensis iriomotensis*) from Japan. Curiously, this last nymph was also found positive to *H. felis* [22].

*Ehrlichia canis* is the etiological agent of canine monocytic ehrlichiosis and its confirmed vectors are *Rhipicephalus sanguineus* (*sensu lato*) ticks [23]. Dogs infected with *E. canis* can present a wide spectrum of clinical conditions, from subclinical infection to fatal illness [24]. *Ehrlichia canis* can also infect cats [25, 26], and human infections of a specific *E. canis* strain have been reported from Venezuela [27]. Most clinical manifestations attributed to canine ehrlichiosis have also been described in infected cats [23, 26].

In general, feline *Hepatozoon* infections are mostly caused by *H. felis*, which has tropism to the myocardial and skeletal muscle tissues of cats, and seems to cause mostly subclinical infection [15]. Nevertheless, non-healthy cats from Cyprus were described as three times more likely to be infected with *Hepatozoon* spp. compared with healthy ones [3]. Among wild felids, *H. felis* was detected at a frequency of around 10% in captive African lions (*Panthera leo*) from Zimbabwe [8]. In southern Italy, apart from *H. felis*, domestic cats have also been found singly infected with *H. canis* and *Hepatozoon silvestris* [28]. The vectors and routes of transmission of *H. felis* are currently not known.

Even though it was not found in the present investigation, *Babesia felis* is an agent of clinical babesiosis among domestic cats in South Africa [7]. Based on reverse line blot (RLB) hybridization, *Babesia leo*, which was originally reported from African lions, was also detected in coinfection with *B. felis* in one domestic cat from this same country [29]. In addition, *Babesia lengau*, first described in cheetahs (*Acinonyx jubatus*), was incriminated as the etiological agent in two severe clinical cases in domestic cats also from South Africa [30].

Another recent molecular study of tick-borne pathogens in dogs (*n* = 103) from Luanda revealed that 20.4% of the dogs were infected with *A. platys*, 17.5% with *H. canis*, 5.8% with *E. canis*, 5.8% with *B. vogeli*, 1.0% with *Babesia gibsoni* and 1.0% with an unnamed *Babesia* sp. [31]. Almost 45% of the dogs were positive to at least one pathogen, which represents a statistically significant difference (CST: *χ*^2^ = 6.38, *df* = 1, *P* < 0.0001) to the 5.9% found positive to at least one agent in cats in the present report. The percent levels of canine single (37.9%) and co-infections (6.8%) also represent significant differences (CST: *χ*^2^ = 5.75, *df* = 1; *P* < 0.0001; and FET: *P* = 0.032) to their corresponding feline values (i.e. 4.9 and 1.0%, respectively). On the other hand, the difference between the molecular prevalence of canine (5.8%) and feline (2.9%) *E. canis* infections was not statistically significant (FET: *P* = 0.314). Lastly, in the present report, *H. felis* was detected at a 2.9% molecular prevalence, which significantly differs (FET: *P* = 0.0006) from the 17.5% of *H. canis* previously found in the domestic dogs from Luanda [31].

The significantly (CST: *χ*^2^ = 6.38, *df* = 1, *P* < 0.0001) higher prevalence of tick-borne pathogens among dogs (44.7%) from Luanda compared with cats (5.9%) may be due to a more frequent exposure of the canine hosts to ticks. In fact, tick infestation in the study in dogs was 60.2%, which is also significantly different (FET: *P* < 0.0001) from the absence of ticks detected on cats (data not shown). Prevention of tick-borne infections in cats largely relies on the regular and long-lasting application of effective acaricide products on individual animals for vector control. Fipronil, macrocyclic lactones, flumethrin and isoxazolines, administered individually or in combination, are among the active ingredients available on the global market for the control of tick infestations in cats [32–34].

The sampled domestic cats might not accurately represent the overall feline population of both Luanda and Angola, and additional studies also including potential vector ticks are needed for a more comprehensive clinical and epidemiological assessment. Under this circumstance, the present preliminary and geographically localized study may have limited the detection of a higher prevalence and even a wider variety of tick-borne agents [35]. Concerning other vector-borne pathogens, all the cats assessed in the present study were found negative for immunoglobulin G antibodies to *Leishmania* spp. by the DAT (direct agglutination test) [36].

## Conclusions

In conclusion, this is the first report of *A. bovis*, *E. canis* and *H. felis* in felids from Luanda in Angola, where domestic cats are exposed to a low to moderate risk of becoming infected with tick-borne pathogens. Additional research is necessary, including a larger number of animals and feline populations from other cities and provinces, aiming at better characterizing and controlling feline vector-borne pathogens and their arthropod vectors in Angola.

## Additional file

Additional file 1: **Table S1.** DNA sequences of the *16S* rRNA gene from *Ehrlichia* spp. and *Anaplasma* spp. and *18S* rRNA gene of *Hepatozoon* spp. amplified from cats positive to tick-borne pathogens. (DOCX 81 kb)

## Abbreviations

95% CI: 95% confidence interval
CST: Chi-square test
FET: Fisher’s exact test
FIV: Feline immunodeficiency virus
PCR: Polymerase chain reaction
rRNA: Ribosomal RNA
