# Supplementary material for: Molecular detection of Anaplasma bovis, Ehrlichia canis and Hepatozoon felis in cats from Luanda, Angola
Source: Parasit Vectors. 2018 Mar 20;11:167. doi: 10.1186/s13071-018-2767-y (PMC5859530; doi:10.1186/s13071-018-2767-y)
Supplement: Supplementary file 1 — Table S1. DNA sequences of the 16S rRNA gene from Ehrlichia spp. and Anaplasma spp. and 18S rRNA gene of Hepatozoon spp. amplified from cats positive to tick-borne pathogens. (DOCX 81 kb) [file 13071_2018_2767_MOESM1_ESM.docx]

**Additional file 1: Table S1.** DNA sequences of the *16S* rRNA gene from *Ehrlichia* spp. and *Anaplasma* spp. and *18S* rRNA gene of *Hepatozoon* spp. amplified from cats positive to tick-borne pathogens

| Pathogen | Sample’s code | Base pairs (bp) | Partial sequence | New GenBank accession |
| --- | --- | --- | --- | --- |
| *Anaplasma bovis* | 026 | 305 | CGGCAAACTCCGTGCCAGCAGCCGCGGTAATACGGCGGGGGCAAGCGTTGTTCGGAATTATTGGGCGTAAAGGGCATGTAGGCGGTCTAGTTAGTTAGAGGTGAAATGCCAGGGCTTAACCCTGGAGCTGCTTTTAATACTGCTAGACTGGAGTCCGGGAGAGGATAGCGGAATTCCTAGTGTAGAGGTGAAATTCGTAGATATTAGGAGGAACACCAGTGGCGAAGGCGGCTATCTGGTCCGGTACTGACGCTGAGGTGCGAAAGCGTGGGGAGCAAACAGGATTAGATACCCTGGTAGTCCAC | MG431981 |
| *Ehrlichia canis* | 002 | 82 | TCCAGTGTGGGCTGATCGTCCTCTCAGACCAGCTATAGATCATAGCCTTGGTAAGCCATTACCTTACCAACTAGCTAATCTA | na |
| *Ehrlichia canis* | 017 | 81 | TCCAGTGTGGGCTGATCGTCCTCTCAGACCAGCTATAGATCATAGCCTTGGTAAGCCTTACCTTACCAACTAGCTAATCTA | na |
| *Anaplasma* sp. (suspected as *Anaplasma* *bovis* and verified by sequencing of a longer *16S* rRNA gene segment shown above) | 026 | 82 | TCCAGTGTGGGCTGATCGTCCTCTCAGACCAGCTATAGATCATAGCCTTGGTAAGCCATTACCTTACCAACTAGCTAATCTA | na |
| *Ehrlichia canis* | 053 | 81 | TCCAGTGTGGCTGATCGTCCTCTCAGACCAGCTATAGATCATAGCCTTGGTAAGCCATTACCTTACCAACTAGCTAATCTA | na |
| *Hepatozoon felis* | 002 | 337 | CAGCTCCAATAGCGTATATTAAAATTGTTGCAGTTAAAAAGCTCGTAGTTGAATTTCTGCTAAAAATAACCGGTCTGCTTTTAATAAAGGTGGTATCTTGGTGTGTTTTTAGCAATAATGTCCTTTGAAATGTTTTTTACTTCATTGTAATAAATTATATTCAGGATTTTTACTTTGAGAAAATTAGAGTGTTTCTAGCAGGCTAATGCTTTGAATACTGCAGCATGGAATAATAAAATAGGATTTTGGTTCTACATTATTGGTTTTAAGAGCTAAATTAATGATTGATAGGGACAGTTGGGGGCATTTGTATTTAACTGTCAGAGGTGAAATTCTT | MG386482 |
| *Hepatozoon felis* | 056 | 337 | CAGCTCCAATAGCGTATATTAAAATTGTTGCAGTTAAAAAGCTCGTAGTTGAATTTCTGCTAAAAATAACCGGTCTGCTTTTAATAAAGGTGGTATCTTGGTGTGTTTTTAGCAATAATGTCCTTTGAAATGTTTTTTACTTCATTGTAATAAATTATATTCAGGATTTTTACTTTGAGAAAATTAGAGTGTTTCTAGCAGGCTAACGCTTTGAATACTGCAGCATGGAATAATAAAATAGGATTTTGGTTCTACATTATTGGTTTTAAGAGCTAAATTAATGATTGATAGGGACAGTTGGGGGCATTTGTATTTAACTGTCAGAGGTGAAATTCTT | MG386483 |
| *Hepatozoon felis* | 063 | 337 | CAGCTCCAATAGCGTATATTAAAATTGTTGCAGTTAAAAAGCTCGTAGTTGAATTTCTGCTAAAAATAACCGGTCTGCTTTTAATAAAGGTGGTATCTTGGTGTGTTTTTAGCAATAATGTCCTTTGAAATGTTTTTTACTTCATTGTAATAAATTATATTTAGGATTTTTACTTTGAGAAAATTAGAGTGTTTCTAGCAGGCTAATGCTTTGAATACTGCAGCATGGAATAATAAAATAGGATTTTGGTTCTACATTATTGGTTTTAAGAGCTAAATTAATGATTGATAGGGACAGTTGGGGGCATTTGTATTTAACTGTCAGAGGTGAAATTCTT | MG386484 |

*Abbreviations*: na, not available, as sequences < 200 bp cannot be deposited in GenBank
